# Automobile Exhaust Removal Performance of Pervious Concrete with Nano TiO_2_ under Photocatalysis

**DOI:** 10.3390/nano10102088

**Published:** 2020-10-21

**Authors:** Guobao Luo, Hanbing Liu, Wenjun Li, Xiang Lyu

**Affiliations:** 1 State Key Laboratory of Mechanical Behavior and System Safety of Traffic Engineering Structures, Shijiazhuang Tiedao University, Shijiazhuang 050043, China; luogb@stdu.edu.cn; 2 Key Laboratory of Roads and Railway Engineering Safety Control (Shijiazhuang Tiedao University), Ministry of Education, Shijiazhuang 050043, China; 3 College of Transportation, Jilin University, Changchun 130025, China; lhb@jlu.edu.cn (H.L.); lvxiang18@mails.jlu.edu.cn (X.L.)

**Keywords:** photocatalysis, nano TiO_2_, ESEM, nitrogen oxides, hydrocarbons, pervious concrete

## Abstract

The urban environment is facing serious problems caused by automobile exhaust pollution, which has led to a great impact on human health and climate, and aroused widespread concern of the government and the public. Nano titanium dioxide (TiO_2_), as a photocatalyst, can be activated by ultraviolet irradiation and then form a strong REDOX potential on the surface of the nano TiO_2_ particles. The REDOX potential can degrade the automobile exhaust, such as nitrogen oxides (NO_x_) and hydrocarbons (HC). In this paper, a photocatalytic environmentally friendly pervious concrete (PEFPC) was manufactured by spraying nano TiO_2_ on the surface of it and the photocatalytic performance of PEFPC was researched. The nano TiO_2_ particle size, TiO_2_ dosage, TiO_2_ spraying amount, and dispersant dosage were selected as factors to investigate the efficiency of photocatalytic degradation of automobile exhaust by PEFPC. Moreover, the environmental scanning electron microscope (ESEM) was used to evaluate the distribution of nano TiO_2_ on the surface of the pervious concrete, the distribution area of nano TiO_2_ was obtained through Image-Pro Plus, and the area ratio of nano TiO_2_ to the surface of the pervious concrete was calculated. The results showed that the recommended nano TiO_2_ particle size is 25 nm. The optimum TiO_2_ dosage was 10% and the optimum dispersant dosage was 5.0%. The photocatalytic performance of PEFPC was best when the TiO_2_ spraying amount was 333.3 g/m^2^. The change in the photocatalytic ratio of HC and NO_x_ is consistent with the distribution area of nano TiO_2_ on the surface of the pervious concrete. In addition, the photocatalytic performance of PEFPC under two light sources (ultraviolet light and sunlight) was compared. The results indicated that both light sources were able to stimulate the photocatalytic performance of PEFPC. The research provided a reference for the evaluation of automobile exhaust removal performance of PEFPC.

## 1. Introduction

In recent decades, global urbanization and development of the automotive industry have caused many environmental problems while promoting rapid social development [1,2]. Urban air pollution, as the most serious one, has had a great impact on urban environment and human health [3]. Automobile exhaust caused by the increasing number of motor vehicles in the city is the main source of urban air pollution. The main components of automobile exhaust are nitrogen oxides (NO_x_) and hydrocarbons (HC). Sustainability development, especially environmental sustainability development, has become a key issue of social development and aroused widespread concern of the government and the public [4]. Automobile exhaust leads to air pollution throughout the world. There are many emission reduction methods, such as the encouragement of carpooling and public transportation, but automobile exhaust pollution is still a serious problem all over the world [5].

Nano titanium dioxide (TiO_2_), characterized by low toxicity, strong optical absorption, and redox ability, and is promising in photocatalytic degradation of automobile exhaust [6,7]. Moreover, nano TiO_2_ has a wide band gap of about 3.2 eV and has significant performance in the ultraviolet light region [8], and automobile exhaust photocatalytic products are harmless to the urban environment [9,10,11]. In recent years, the rapid development of TiO_2_ photocatalytic technology has prompted the application of photocatalytic pavement materials to improve urban air quality [12,13]. Nano TiO_2_ is the most extensively used photocatalyst due to its low cost and high chemical stability [14,15]. Pervious concrete is considered to be an environmentally friendly pavement material, which has many environmental benefits, such as improving water quality and reducing soil pollution. The use of pervious concrete meets the requirements of sustainable social development [16]. The incorporation of nano TiO_2_ into pervious concrete pavement materials imparts photocatalytic performance to pervious concrete [17,18,19]; automobile exhaust nearby pervious concrete pavement can be converted to water and salt under ultraviolet light irradiation and subsequently the salt will be washed away by the rain [20,21].

Nano TiO_2_ exists in three different crystalline forms: anatase, rutile, and brookite [22]. Anatase TiO_2_ has an indirect bandgap while rutile has a direct bandgap. The ability of indirect bandgap to inhibit the recombination of the electron and hole is better than that of direct bandgap, which results in anatase TiO_2_, which is better than rutile TiO_2_ in photocatalytic activity [23]. It is difficult to produce brookite TiO_2_ in the laboratory due to its intermediate phase in the anatase to brookite to rutile [24]. Under ultraviolet irradiation, electrons in the valence band are activated and transferred into the conduction band, leaving relatively stable holes on the surface of the valence band, thus forming electron–hole pairs. The electron and hole are captured by imperfection and dangling bond of nano TiO_2_ and diffuse to the surface of nano TiO_2_, forming a strong REDOX potential and degrading automobile exhaust into harmless substances. For various reasons, anatase TiO_2_ is the preferred photocatalyst due to its strong photocatalytic activity, simple preparation and the absence of toxicity [18,23].

In recent years, many experiments have been performed under different experimental conditions in order to evaluate the photocatalytic performance of nano TiO_2_ and promote the application of photocatalytic pavement materials. Some scholars have studied the effects of substrate materials on the photocatalytic performance of nano TiO_2_. Xu et al. [25] studied the effect of recycled aggregate on the photocatalytic performance of nano TiO_2_ concrete. The results showed that the recycled aggregate coated with nano TiO_2_ significantly improved the photocatalytic performance of nano TiO_2_ concrete. Chen et al. [13] used recycled glass to replace sand in preparing the concrete; the effects of glass color, aggregate size, and curing age on the photocatalytic performance of nano TiO_2_ concrete were investigated. The significant improvement of photocatalytic performance was obtained due to the use of recycled glass in the concrete. Guo et al. [12] evaluated the effects of white cement and ground granulated blast furnace slag on the photocatalytic performance of nano TiO_2_ concrete. The results showed that the use of white cement and ground granulated blast furnace slag had a positive effect on photocatalytic performance of nano TiO_2_ concrete. Some scholars have studied the effect of environmental factors on photocatalytic performance of nano TiO_2_. Guo et al. [26] compared the effects of different NO flow rates, initial NO concentrations, ultraviolet light intensities, and relative humidity conditions on the photocatalytic performance of nano TiO_2_ concrete. Ballari et al. [18] studied the effects of initial NO_x_ concentration, reactor height, and flow rate on the photocatalytic performance of nano TiO_2_ concrete and proposed the corresponding kinetic model. However, research about the photocatalytic degradation of HC is not enough, and even fewer studies were reported on the effect of dispersion of TiO_2_ on photocatalytic performance.

**Figure 1:**
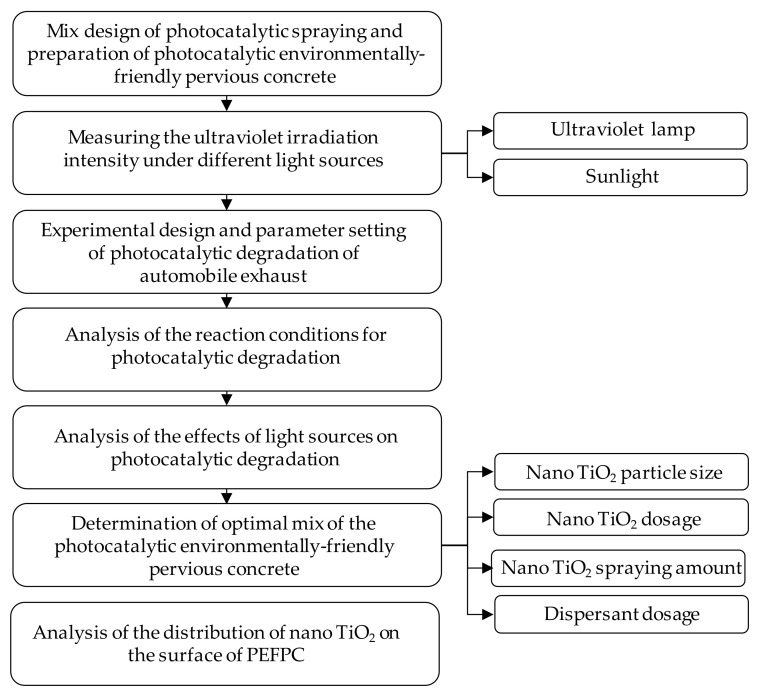
Research outline.

In this study, in allusion to the problem of excessive automobile exhaust, the photocatalytic performance of PEFPC was evaluated. The TiO_2_ particle size, TiO_2_ dosage, TiO_2_ spraying amount and dispersant dosage were selected as effect factors and the experiment of photocatalytic performance of PEFPC was conducted, and the distribution of nano TiO_2_ on the surface of PEFPC was evaluated by the environmental scanning electron microscope (ESEM). Moreover, the photocatalytic performance of PEFPC under two light sources (ultraviolet light and sunlight) was compared and the application of PEFPC was evaluated. The research outline of the study is shown in Figure 1.

## 2. Photocatalytic Degradation Mechanism

Ultraviolet light irradiates the surface of nano TiO_2_, and the photons in the ultraviolet light are captured by nano TiO_2_. The photons with an energy equal to or greater than the band gap can activate the electrons in the valence band of nano TiO_2_. The activated electrons jump into the conduction band, thus resulting in the generation of holes in the valence band. The electron–hole pairs may rapidly recombine and release energy, or are captured by the surface of nano TiO_2_ to form a REDOX potential. The electrons and holes have strong reducibility and strong oxidability, respectively. The holes react with water to form strong oxidizing hydroxyl radicals. The electrons react with oxygen to form superoxide anions, which have strong REDOX properties. NO_x_ is oxidized to nitrate by hydroxyl radicals and superoxide anions. HC is oxidized and reduced to water and carbon dioxide by hydroxyl radicals and superoxide anions [27,28]. It is the basis condition for photocatalytic reaction that nano TiO_2_ molecules capture a sufficient number and energy of photons. The number of captured photons is related to the contact area of nano TiO_2_ molecules and ultraviolet light. The contact area was affected by TiO_2_ particle size, TiO_2_ dosage, TiO_2_ spraying amount, and dispersant dosage. The energy of photons is related to the intensity of ultraviolet light. In this study, the effects of contact area of nano TiO_2_ molecules and ultraviolet light and the intensity of ultraviolet light on the photocatalytic degradation HC and NO_x_ were investigated.

## 3. Materials and Methods

### 3.1. Materials

In all experiments, Type P.O Portland cement (Changchun, China) with strength grade of 42.5 MPa was selected as the cementitious materials. The chemical composition of cement is shown Table 1. The natural aggregate with particle of 4.75–9.5 mm was used as coarse aggregate to prepare pervious concrete. The water reducer used was liquid polycarboxylic acid series superplasticizer with effective content of 22% and water-reducing ratio of 25%. An anatase nano TiO_2_ obtained from Chengdu Oenris Chemical Reagent Co., Ltd. (Chengdu, China) was used as photocatalyst. The particle size of the TiO_2_ is 10 nm (5–15 nm), 25 nm (20–30 nm), and 50 nm, and its purity is 99%. The sodium hexametaphosphate ((NaPO_3_)_6_) obtained from Chengdu Oenris Chemical Reagent Co., Ltd. (Chengdu, China) was used as dispersant. The sodium hexametaphosphate is analytically reagent and its purity is 65–70%.

**Figure 2:**
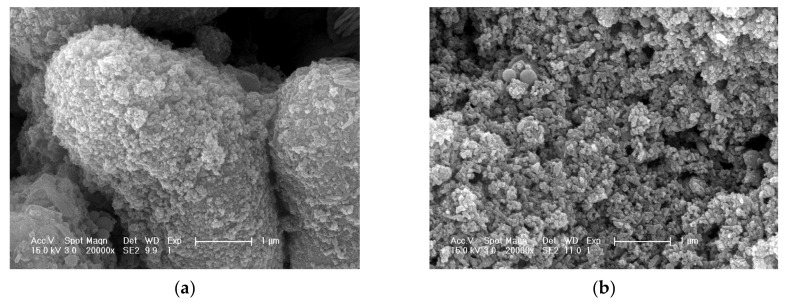
Nano titanium dioxide (TiO_2_): (**a**) before adding dispersant; (**b**) after adding dispersant.

The VDW (Van der Waals’ force) and coulomb force exist in between nano TiO_2_ particles, which leads to the particles absorbing and aggregating with each other, thus losing the dispersion stability of the particles in the suspension. The aggregated nano TiO_2_ particles are shown in Figure 2a. Sodium hexametaphosphate can form a stable complex with nano TiO_2_ molecules. The complex increases the Zeta potential on the surface of the nano TiO_2_ particles and leads to the increase in the repulsive force between nano TiO_2_ particles, thus improving the dispersion stability of nano TiO_2_ particles in the suspension [29]; the nano TiO_2_ particles after adding dispersant are shown in Figure 2b.

### 3.2. Preparation of the Photocatalytic Environmentally Friendly Pervious Concrete

The volumetric method was used for the mixture design of pervious concrete according to the Chinese national standard CJJ/T135-2009 [30]. Based on the previous research conducted by our group [31,32], pervious concrete with optimal mixing ratio was used as substrate materials in this paper, the permeability and effective porosity reached above 3.90 mm/s and 14%, respectively, and the compressive strength was 24 MPa. Moreover, pervious concrete specimens were made using the cement paste encapsulating aggregate method and rodding method. The water–binder ratio and porosity were 0.30 and 15%, respectively. The amount of water reducer was 0.5% of the mass of cement. The specimen size was 300 × 300 × 50 mm. All the specimens were demolded after 24 h and placed in standard-cured room with a relative humidity of 95% and a temperature of 20 ± 2 °C for 28 days. Nano TiO_2_ and sodium hexametaphosphate were dispersed into water to prepare TiO_2_ coating, and then the TiO_2_ coating was sprayed on the surface of pervious concrete and tested after natural air drying for 24 h. The dispersant dosage is the mass percentage of nano TiO_2_. The mixing ratio of photocatalytic coating is shown in Table 2.

### 3.3. Determination of Light Intensity

**Figure 3:**
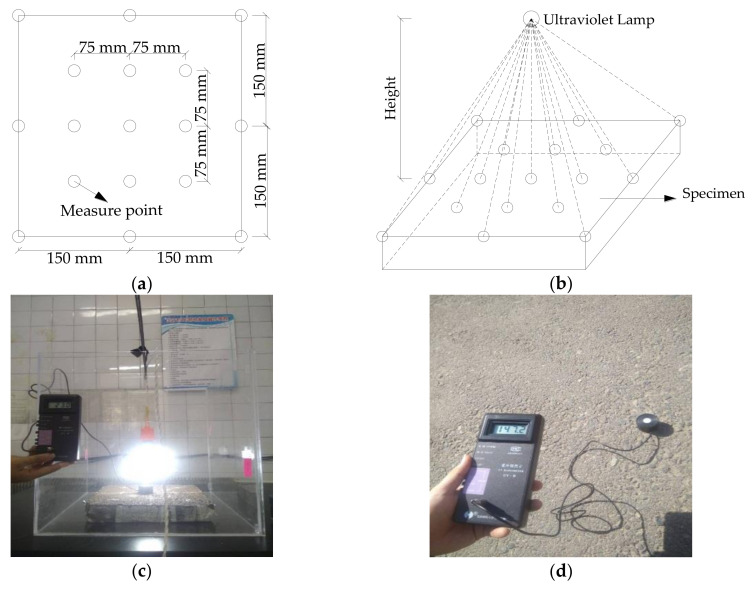
Ultraviolet irradiation intensity experiment of ultraviolet lamp and sunlight: (**a**) measuring point diagram; (**b**) test diagram; (**c**) ultraviolet intensity test; (**d**) sunlight intensity test.

Nano TiO_2_ can be motivated to produce electrons and holes under ultraviolet irradiation. An ultraviolet lamp was used as the experimental light source and the ultraviolet wavelength is 285–297 nm. 17 measurement points on the surface of the specimen were chosen, which are shown in Figure 3a. The ultraviolet lamp and specimen were placed in the middle of the reactor. The ultraviolet irradiance meter was used to measure the ultraviolet irradiation intensity of the measured points at different ultraviolet lamp heights. Just as shown in Figure 3b,c, the ultraviolet lamp was placed directly above the specimen and the distance between the ultraviolet lamp and the center of the specimen was 135 mm, 180 mm, 225 mm, and 270 mm, respectively. The average ultraviolet irradiation intensity at different ultraviolet lamp heights was calculated and the results are shown in Table 3. The ultraviolet irradiation intensity on the hour of the summer solstice was measured, which is shown in Figure 3d. The average ultraviolet irradiation intensity of the day was calculated, and the result is shown in Table 3.

### 3.4. Photocatalytic Degradation Experiment

**Figure 4:**
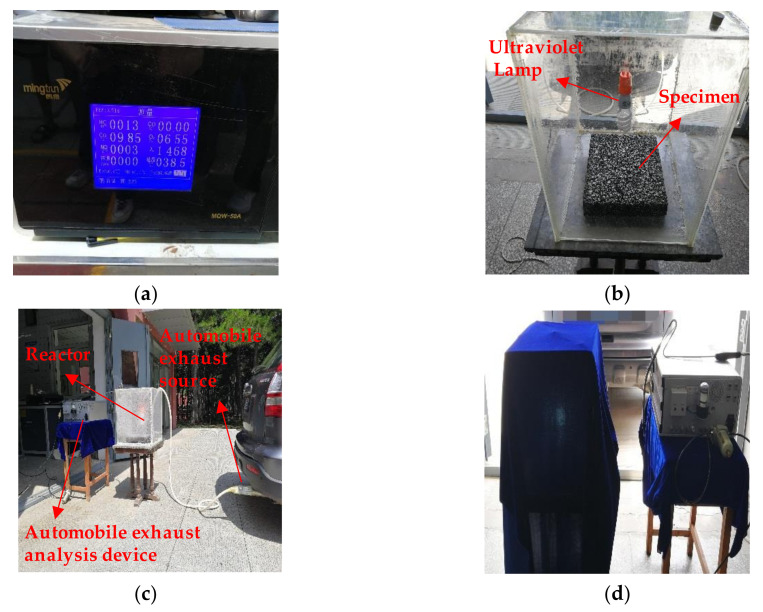
The apparatus of photocatalytic degradation experiment: (**a**) automobile exhaust analysis device; (**b**) reactor; (**c**) test device; (**d**) test process.

The experimental device consisted of three parts: automobile exhaust analysis device, reactor, and automobile exhaust source, as shown in Figure 4a–c. The reactor was completely sealed and had a good air tightness. The size of reactor is 450 × 450 × 500 mm. MQW-50A tail gas analyzer obtained from Zhejiang University Mingquan Electronic Technology Co., Ltd. (Hangzhou, China) was used to analyze the automobile exhaust components. The measurement range of NO_x_ and HC was 0–5000 ppm and 0–10,000 ppm respectively and the relative measurement error was 4% and 3%, respectively. Both NO_x_ and HC had a resolution of 1 ppm. The experimental procedures are as follows: (1) put the specimen into the bottom middle position of the reactor and adjust the height of the ultraviolet lamp as shown in Figure 4b; (2) connect the automobile exhaust source to the reactor with a rubber hose as shown in Figure 4c; (3) add automobile exhaust into the reactor and turn on the automobile exhaust analysis device for real-time monitoring until the initial concentrations of NO_x_ and HC are reached; (4) cover the reactor with a shading cloth to prevent interference with natural light and then turn on the ultraviolet lamp for the test as shown in Figure 4d. All experiments were performed at ambient temperature and one atmosphere. The concentrations of NO_x_ and HC in the actual traffic jam section are about 15 ppm and 35 ppm, respectively. Therefore, the initial concentrations of NO_x_ and HC in this experiment were set as 80 ppm and 160 ppm, respectively. The reaction time was 60 min, and the NO_x_ and HC concentrations in the reactor were tested every 10 min. The photocatalytic degradation ratios of NO_x_ and HC on the surface of PEFPC are calculated as Equation (1). The photocatalytic degradation rates of NO_x_ and HC on the surface of PEFPC are calculated as Equation (2).

(1) $$D=\frac{G_{intial}-G_{final}}{G_{intial}}\times 100\%$$

(2) $$F=\frac{G_{intialN}-G_{finalN}}{T}$$

where *D* is the photocatalytic degradation ratio of NO_x_ and HC (%); *G_intial_* is the initial concentration of NO_x_ and HC (ppm); *G_final_* is the final concentration of NO_x_ and HC (ppm); *F* is the photocatalytic degradation rate of NO_x_ and HC (ppm/min); *G_intialN_* is the initial concentration of NO_x_ and HC in the *N*th ten minutes (ppm); *G_finalN_* is the final concentration of NO_x_ and HC in the *N*th ten min (ppm); T is the reaction time and T = 10 min.

### 3.5. ESEM

**Figure 5:**
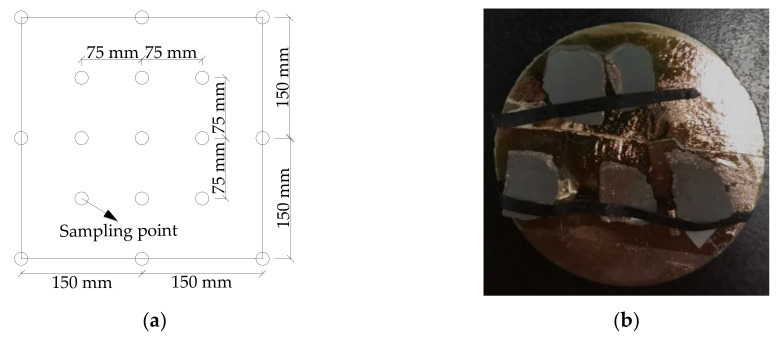
The sampling diagram and processed samples: (**a**) schematic diagram; (**b**) processed samples.

The efficiency of photocatalytic automobile exhaust degradation is related to the contact area of nano TiO_2_ and ultraviolet light. In this paper, the ESEM was adopted to obtain the microscopic characterization of nano TiO_2_. 17 points (shown in Figure 5a) on the surface of pervious concrete sample were selected as sampling points, and among them, 3 evenly selected measuring points were chosen for taking pictures. The samples were air-dried and gilded, and it should be noted that the observation surface of the concrete sample should not be damaged during the processing of the sample. The processed sample is shown in Figure 5b.

**Figure 6:**
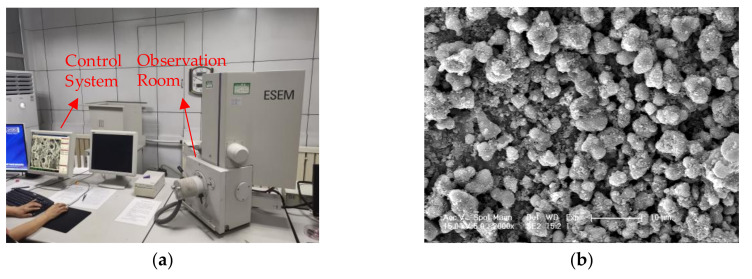
The environmental scanning electron microscope (ESEM) equipment and sample microscopic picture: (**a**) ESEM equipment; (**b**) sample microscopic picture.

The processed sample was put into the ESEM observation room, the ESEM equipment is shown in Figure 6a, the sample on the worktable was leveled by silicon wafers or other dry materials to avoid errors caused during imaging. The picture taken by ESEM is shown in Figure 6b.

The Image-Pro Plus (Rockville, MD, USA) was used to process the picture and extract the area value of nano TiO_2_ in the picture, and the area ratio of nano TiO_2_ area to the measuring point area was calculated and the average value of 51 measuring points were adopted to characterize the contact area of nano TiO_2_ and ultraviolet light. The specific calculation is shown in Equation (3).

(3) $$Area\;Ratio=\frac{\sum_{1}^{51}\frac{Area_{T}}{Area_{mp}}}{51}$$

where *Area Ratio* is the average value of 51 measuring points to characterize the ratio of nano TiO_2_ area to the pervious concrete substrate materials; *Area_T_* is the nano TiO_2_ area of the measuring point; *Area_mp_* is the area of measuring point.

## 4. Results

### 4.1. The Reaction Conditions for Photocatalytic Degradation

**Figure 7:**
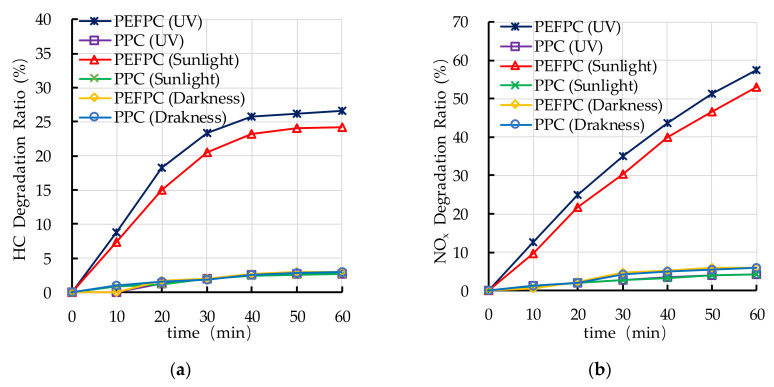
Effect of photocatalyst and light source on photocatalytic degradation of hydrocarbons (HC) and nitrogen oxides (NO_x_): (**a**) HC degradation ratio; (**b**) NO_x_ degradation ratio.

The experimental results of photocatalytic degradation of HC and NO_x_ by PEFPC and plain pervious concrete (PPC) with different light sources are shown in Figure 7. It can be seen from Figure 7 that the PPC cannot significantly photocatalytic degrade HC and NO_x_, and only a small amount of HC and NO_x_ were consumed by PEFPC and PPC with dark conditions, while the PEFPC can significantly photocatalytically degrade HC and NO_x_ under the ultraviolet light irradiation condition. It can be concluded that photocatalyst and ultraviolet irradiation are the basic conditions for photocatalytic degradation of HC and NO_x_. Figure 7 shows that the PEFPC can significantly photocatalytically degrade HC and NO_x_ under the sunlight irradiation condition. It indicates that sunlight can stimulate the activity of nano TiO_2_ on the surface of PEFPC and meet the basic requirements of photocatalytic reaction.

### 4.2. Effect of Light Source on Photocatalytic Degradation

**Figure 8:**
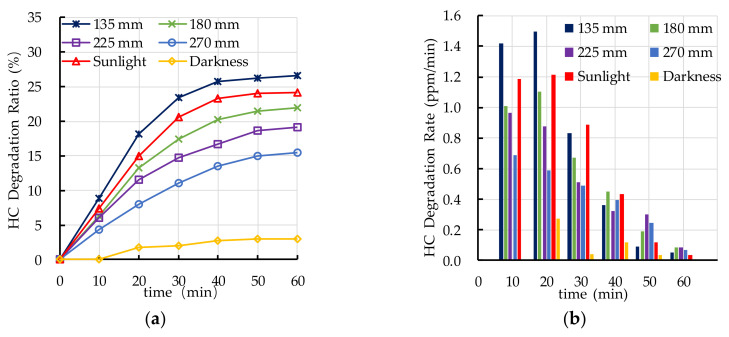
Effect of light source on photocatalytic degradation of HC: (**a**) HC degradation ratio; (**b**) HC degradation rate.

Ultraviolet irradiation is needed as an elementary condition for the photocatalytic degradation reaction. In this section, the effects of different light sources on photocatalytic degradation of automobile exhaust of PEFPC was studied. The selected light sources are ultraviolet height 135 mm, ultraviolet height 180 mm, ultraviolet height 225 mm, ultraviolet height 270 mm, sunlight, and darkness. The experimental results of photocatalytic degradation of HC are shown in Figure 8. It can be seen from Figure 8 that the photocatalytic degradation ratio of HC increases when the ultraviolet irradiation intensity increases from 31.8 μW/cm^2^ to 105.1 μW/cm^2^. The photocatalytic degradation ratio and rate of HC under ultraviolet light irradiation with a lamp height of 135 mm are similar to that under sunlight irradiation. The photocatalytic degradation ratios and rates of HC of ultraviolet height 180 mm, ultraviolet height 225 mm, and ultraviolet height 270 mm are lower than that of ultraviolet height 135 mm and sunlight.

**Figure 9:**
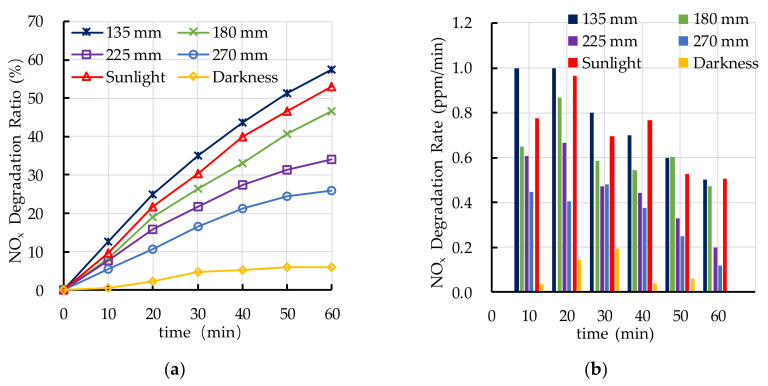
Effect of light source on photocatalytic degradation of NO_x_: (**a**) NO_x_ degradation ratio; (**b**) NO_x_ degradation rate.

The experimental results of photocatalytic degradation of NO_x_ are shown in Figure 9. It can be seen that the photocatalytic degradation ratio of NO_x_ increases with the increasing in ultraviolet light irradiation intensity. The photocatalytic degradation ratio and rate of NO_x_ under ultraviolet light irradiation with a lamp height of 135 mm are similar to that under sunlight irradiation. The photocatalytic degradation ratios and rates of NO_x_ of ultraviolet lamp 180 mm, ultraviolet lamp 225 mm, and ultraviolet lamp 270 mm are lower than that of ultraviolet lamp 135 mm and sunlight. The experimental height of ultraviolet lamp in the following experiment is selected as 135 mm for the reason that the experimental results of photocatalytic degradation of HC and NO_x_ under ultraviolet light irradiation with a lamp height of 135 mm is similar to that under sunlight irradiation.

### 4.3. Effect of TiO_2_ Particle Size on Photocatalytic Degradation

**Figure 10:**
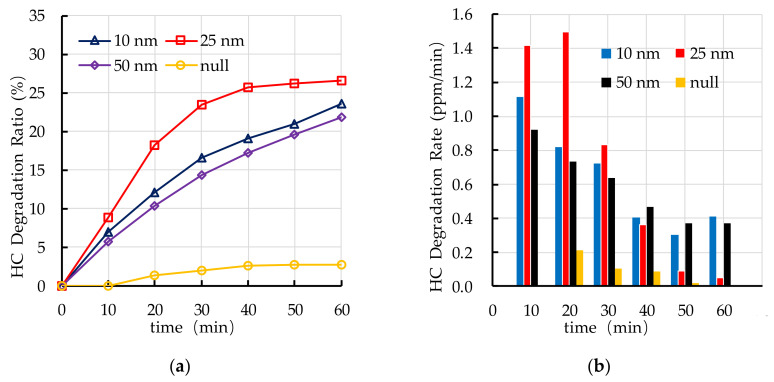
Effect of TiO_2_ particle size on photocatalytic degradation of HC: (**a**) HC degradation ratio; (**b**) HC degradation rate.

The experimental results of photocatalytic degradation of HC by PEFPC with different TiO_2_ particle sizes are shown in Figure 10. The photocatalytic degradation ratio of 25 nm TiO_2_ on degrading HC is better than that of 10 nm and 50 nm. The photocatalytic degradation rate of 25 nm TiO_2_ is higher in the first 30 min and lower in the last 30 min than that of 10 nm and 50 nm, but overall, the photocatalytic degradation ratio of 25 nm is higher than that of 10 nm and 50 nm.

**Figure 11:**
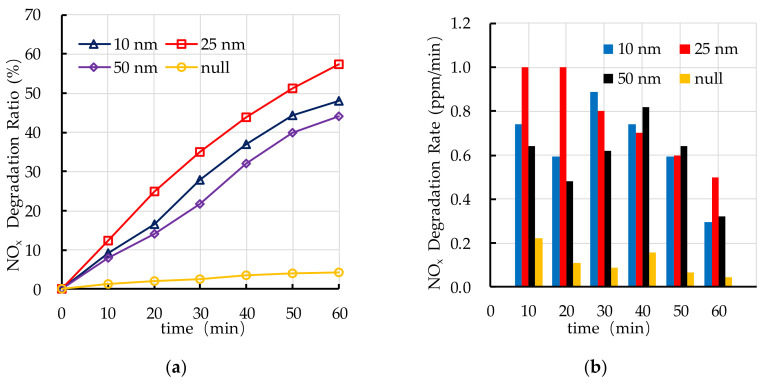
Effect of TiO_2_ particle size on photocatalytic degradation of NO_x_: (**a**) NO_x_ degradation ratio; (**b**) NO_x_ degradation rate.

Figure 11 shows the experimental results of photocatalytic degradation of NO_x_ by PEFPC with different TiO_2_ particle sizes. The photocatalytic degradation ratio of 25 nm TiO_2_ on degrading NO_x_ is the best, followed by 10 nm and 50 nm. The photocatalytic degradation ratio of PEFPC with three particle sizes reaches over 40% after 60 min. In addition, the average photocatalytic degradation rate of NO_x_ of 25 nm TiO_2_ is 0.77 ppm/min, 10 nm TiO_2_ is 0.64 ppm/min and 50 nm TiO_2_ is 0.59 ppm/min.

### 4.4. Effect of TiO_2_ Dosage on Photocatalytic Degradation

**Figure 12:**
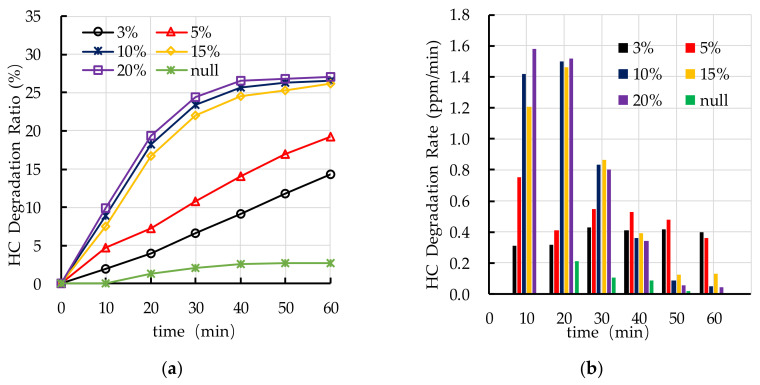
Effect of TiO_2_ dosage on photocatalytic degradation of HC: (**a**) HC degradation ratio; (**b**) HC degradation rate.

The effect of TiO_2_ dosage on photocatalytic degradation of HC is shown in Figure 12. It is found that the TiO_2_ dosage has a great impact on the photocatalytic degradation ratio of HC. When the TiO_2_ dosage is larger than 10%, the photocatalytic degradation ratio of HC is better. The photocatalytic degradation ratio of HC increases with the increase of TiO_2_ dosage (3–10%). When the TiO_2_ dosage is 10–20%, the photocatalytic degradation ratio of HC changes little, which means that the 10% TiO_2_ is enough and it can be used as the critical value for photocatalytic degradation of HC. When the TiO_2_ dosage is 10%, the photocatalytic degradation rate of HC decreases with the increase of reaction time, it indicates that the reaction of photocatalytic degradation of HC mainly occurs in the first 30 min.

**Figure 13:**
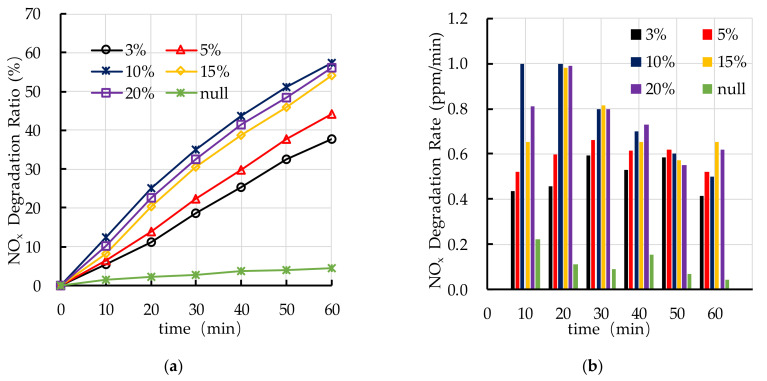
Effect of TiO_2_ dosage on photocatalytic degradation of NO_x_: (**a**) NO_x_ degradation ratio; (**b**) NO_x_ degradation rate.

Figure 13 shows the effect of TiO_2_ dosage on photocatalytic degradation of NO_x_. It can be seen that the photocatalytic degradation ratio of NO_x_ is the highest at 10% TiO_2_ dosage and the photocatalytic degradation ratio reaches 59% at 60 min. When the TiO_2_ dosage is 15% and 20%, the photocatalytic degradation ratio of NO_x_ is similar to that of 10%. The photocatalytic degradation rate of NO_x_ with different TiO_2_ dosages is relatively stable. When the TiO_2_ dosage is 10%, the average photocatalytic degradation rate of NO_x_ is highest, and the average rate is 0.77 ppm/min. It is observed that the photocatalytic degradation of automobile exhaust with 10% TiO_2_ dosage is optimal.

### 4.5. Effect of TiO_2_ Spraying Amount on Photocatalytic Degradation

**Figure 14:**
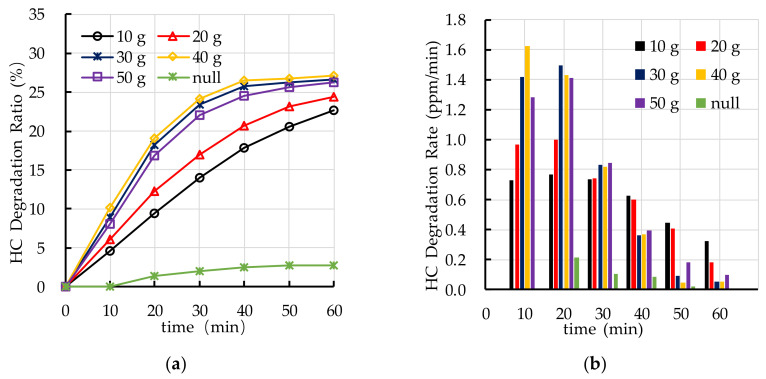
Effect of TiO_2_ spraying amount on photocatalytic degradation of HC: (**a**) HC degradation ratio; (**b**) HC degradation rate.

Figure 14 shows the effects of different TiO_2_ spraying amounts on photocatalytic degradation of HC. Figure 14a shows that the photocatalytic degradation ratio of HC of PEFPC with 30 g, 40 g, and 50 g TiO_2_ spraying amounts is the best. Figure 14b shows that the photocatalytic degradation rate of HC in the first 30 min is higher than that in the last 30 min, it indicates that the photocatalytic activity of nano TiO_2_ on the surface of PEFPC gradually decreases with the increase of reaction time. The reaction of photocatalytic degradation of HC mainly occurs in the first 30 min.

**Figure 15:**
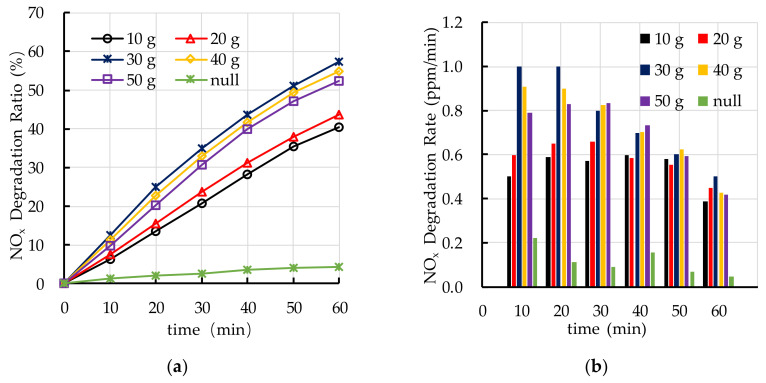
Effect of TiO_2_ spraying amount on photocatalytic degradation of NO_x_: (**a**) NO_x_ degradation ratio; (**b**) NO_x_ degradation rate.

Figure 15 shows the effects of different TiO_2_ spraying amounts on photocatalytic degradation of NO_x_. The results show that the photocatalytic degradation ratio of the specimen with 30 g TiO_2_ spraying amount is the best. It is worth noting that the photocatalytic degradation ratio of NO_x_ of PEFPC increases with the increase of TiO_2_ spraying amounts, when the TiO_2_ spraying amounts is greater than 30 g, the photocatalytic degradation ratio of NO_x_ of PEFPC slightly decrease. In addition, comparing the reaction process of photocatalytic degradation of NO_x_ with that of HC, the photocatalytic degradation rate of NO_x_ is higher than that of HC, which means that photocatalytic degradation of NO_x_ by nano TiO_2_ is more effective than that of HC. Based on the experimental results of photocatalytic degradation of HC and NO_x_, the optimal TiO_2_ spraying amount is 30 g (333.3 g/m^2^).

### 4.6. Effect of Dispersant Dosage on Photocatalytic Degradation

**Figure 16:**
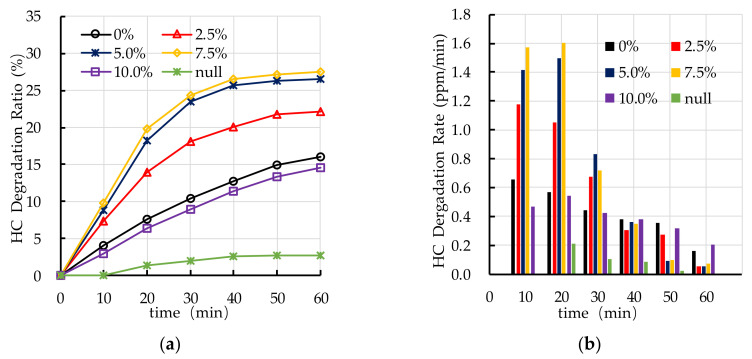
Effect of dispersant dosage on photocatalytic degradation of HC: (**a**) HC degradation ratio; (**b**) HC degradation rate.

Figure 16 shows the experimental results of photocatalytic degradation of HC by different dispersant dosages. The results show that dispersant has a positive effect on photocatalytic degradation of HC. However, excessive dispersant dosage (7.5–10.0%) has a negative effect on photocatalytic degradation of HC. Figure 16a shows that the photocatalytic degradation ratio of HC increases first and then decreases with the increase of dispersant dosage. Figure 16b shows that the photocatalytic degradation rate of HC gradually decreases with the increase of reaction time. The main stage of degradation of HC occurs in the first 30 min.

**Figure 17:**
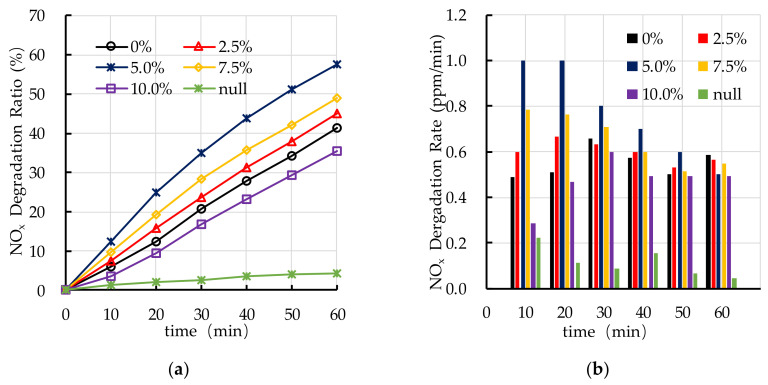
Effect of dispersant dosage on photocatalytic degradation of NO_x_: (**a**) NO_x_ degradation ratio; (**b**) NO_x_ degradation rate.

Figure 17 shows the effects of different dispersant dosages on photocatalytic degradation of NO_x_. The results show that dispersant has a great effect on photocatalytic degradation of NO_x_. It can be observed that the photocatalytic degradation ratio of NO_x_ increases first with the increasing in dispersant dosage (0–5.0%) and then decreases with the increase of dispersant dosage (5.0–10.0%). The photocatalytic degradation rate of NO_x_ is relatively stable, which is different to the degradation of HC. Nano TiO_2_ keeps good photocatalytic activity throughout the whole experiment. According to the experimental results of photocatalytic degradation of HC and NO_x_, the optimal dispersant dosage is 5.0%.

## 5. Discussion

### 5.1. TiO_2_ Particle Size

**Figure 18:**
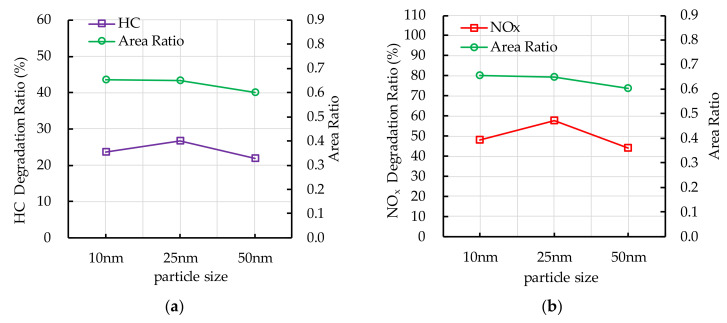
Effect of TiO_2_ particle size on photocatalytic degradation and area ratio: (**a**) HC; (**b**) NO_x_.

The experiment results of photocatalytic degradation reaction and the ESEM by different TiO_2_ particle size are shown in Figure 18. When the photocatalytic degradation reaction is carried out for 60 min, the degradation ratio of HC and NO_x_ first increases and then decreases with the increasing in particle size, while the area ratio gradually decreases as the increasing in particle size. The increase in the area ratio means that the contact area of nano TiO_2_ and ultraviolet light increases, which has a positive effect on the photocatalytic reaction. However, when the TiO_2_ particle size is less than 25 nm, the degradation ratio of HC and NO_x_ increases as the area ratio decreases, which is related to the band gap of nano TiO_2_. When the particle size of nano TiO_2_ is less than 10 nm, the band gap becomes larger, it indicates that the stronger photon energy is needed to activate electrons, which has a negative effect on the photocatalytic reaction [33].

### 5.2. TiO_2_ Dosage

**Figure 19:**
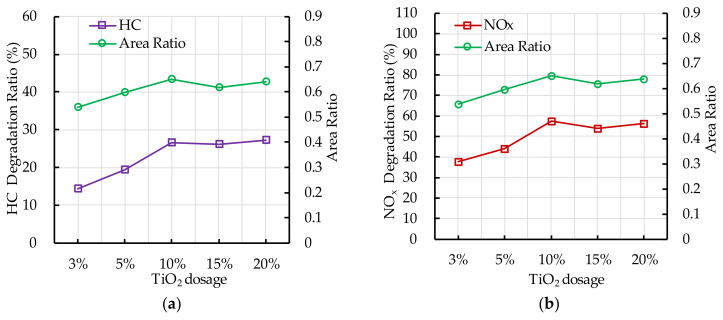
Effect of TiO_2_ dosage on photocatalytic degradation and area ratio: (**a**) HC; (**b**) NO_x_.

Figure 19 shows that the experiment results of photocatalytic degradation reaction and the ESEM by different TiO_2_ dosage. The photocatalytic degradation ratio of HC and NO_x_ at 60 min increases as the nano TiO_2_ dosage increases; while the nano TiO_2_ dosage is 15%, the photocatalytic degradation ratio occurs slight decrease. However, overall, when the nano TiO_2_ dosage is greater than 10%, the photocatalytic degradation ratio changes little. The area ratio has the same trend as the photocatalytic degradation ratio of HC and NO_x_. It indicates that increasing the nano TiO_2_ dosage has a positive effect on improving the contact area of nano TiO_2_ and ultraviolet light. However, excessive nano TiO_2_ particles, namely the nano TiO_2_ dosage is greater than 10%, absorb and aggregate with each other due to the VDW and coulomb force exist in the nano TiO_2_ particles, which results the contact area change little. Therefore, it has little effect on the photocatalytic degradation of HC and NO_x_.

### 5.3. TiO_2_ Spraying Amount

**Figure 20:**
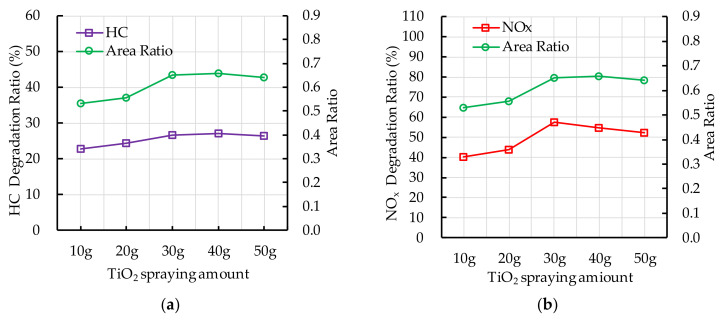
Effect of TiO_2_ spraying amount on photocatalytic degradation and area ratio: (**a**) HC; (**b**) NO_x_.

The experiment results of photocatalytic degradation reaction and the ESEM by different TiO_2_ spraying amount are shown in Figure 20. The photocatalytic degradation ratios of HC and NO_x_ at the end of photocatalytic degradation reaction increase first and then decrease with the increase of nano TiO_2_ spraying amount. The area ratio has the similar trend as the photocatalytic degradation ratio of HC and NO_x_. It indicates that increasing the TiO_2_ spraying amount has a positive effect on improving the contact area of nano TiO_2_ and ultraviolet light. However, the excessive TiO_2_ spraying amount, namely the TiO_2_ spraying amount is greater than 30 g, leads to the nano TiO_2_ particles absorbing and aggregating with each other and spraying film affects the nano TiO_2_ particles absorb ultraviolet light, which results the contact area changes little or even slightly decreases. Therefore, it has slight negative effect on the photocatalytic degradation of HC and NO_x_.

### 5.4. Dispersant Dosage

**Figure 21:**
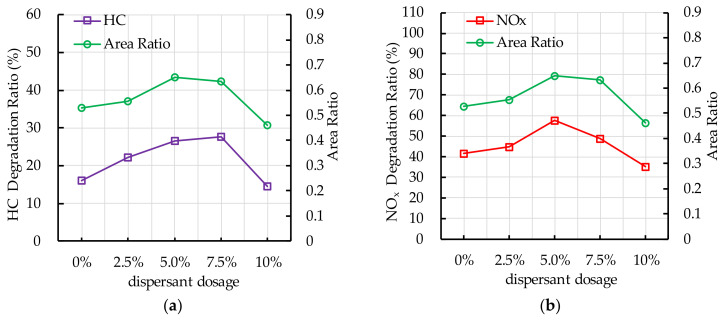
Effect of dispersant dosage on photocatalytic degradation and area ratio: (**a**) HC; (**b**) NO_x_.

Figure 21 shows that the experiment results of photocatalytic degradation reaction and the ESEM by different dispersant dosage. When the photocatalytic degradation reaction lasts for 60 min, the photocatalytic degradation ratio of HC and NO_x_ first increases and then decreases with the increasing in dispersant dosage. The area ratio first increases and then decreases with the increasing in dispersant dosage. The change trend of area ratio is similar to the trend of photocatalytic degradation ratio of HC and NO_x_. Adding dispersant has a positive effect on the dispersion stability of nano TiO_2_ in the TiO_2_ coating, therefore increasing the area ratio of TiO_2_. However, excessive dispersant affects the dispersion stability of nano TiO_2_. When the dispersant dosage reaches 10%, the dispersion stability of nano TiO_2_ is worse than that of coating without dispersant, which has a negative effect on the photocatalytic degradation of HC and NO_x_.

## 6. Conclusions

The photocatalytic performance of PEFPC sprayed with TiO_2_ was prepared and studied in the lab. The effects of light source, TiO_2_ particle size, TiO_2_ dosage, TiO_2_ spraying amount, and dispersant dosage on the photocatalytic degradation were analyzed. Moreover, the distribution of nano TiO_2_ was evaluated by the ESEM. Based on the results, the following conclusions can be drawn:

1. Ultraviolet irradiation is needed as an elementary condition for the photocatalytic degradation reaction. The photocatalytic degradation ratio of automobile exhaust increases with the increase of ultraviolet irradiation intensity. The PEFPC can effectively degrade automobile exhaust and significantly improve urban air quality.
2. The recommend nano TiO_2_ particle size is 25 nm. The most ideal TiO_2_ dosage and dispersant dosage are 10% and 5.0%, respectively. The optimal TiO_2_ spraying amount is 333.3 g/m^2^.
3. The photocatalytic degradation of HC and NO_x_ is different. The photocatalytic degradation of HC can be divided into two stages: rapid stage in the first 30 min and slow stage in the last 30 min. The photocatalytic degradation of NO_x_ is relatively stable.
4. The change in the photocatalytic ratio of PEFPC is consistent with the distribution area of nano TiO_2_ on the surface of the substrate materials. The contact area between nano TiO_2_ and ultraviolet light is a key factor affecting the photocatalytic performance of PEFPC.

## Figures and Tables

**Table 1:** The chemical composition of cement.

Material	Chemical Composition (%)					
	SiO_2_	Al_2_O_3_	Fe_2_O_3_	CaO	MgO	SO_3_
Cement	22.60	5.60	4.30	62.70	1.70	2.50

**Table 2:** Mixing ratio of photocatalytic coating.

Mix ID	TiO_2_ Particle Size (nm)	TiO_2_ Dosage (%)	TiO_2_ (g)	TiO_2_ Spraying Amount ^1^ (g/m^2^)	TiO_2_ Spraying Amount (g)	Dispersant Dosage (%)	Dispersant (g)
PZ1	10	10	3.0	333.3	30	5.0	0.150
PZ2	25	10	3.0	333.3	30	5.0	0.150
PZ3	50	10	3.0	333.3	30	5.0	0.150
D1	25	3	0.9	333.3	30	5.0	0.045
D2	25	5	1.5	333.3	30	5.0	0.075
D3	25	10	3.0	333.3	30	5.0	0.150
D4	25	15	4.5	333.3	30	5.0	0.225
D5	25	20	6.0	333.3	30	5.0	0.300
SD1	25	10	1.0	111.1	10	5.0	0.050
SD2	25	10	2.0	222.2	20	5.0	0.100
SD3	25	10	3.0	333.3	30	5.0	0.150
SD4	25	10	4.0	444.4	40	5.0	0.200
SD5	25	10	5.0	555.5	50	5.0	0.250
DD1	25	10	3.0	333.3	30	0	0
DD2	25	10	3.0	333.3	30	2.5	0.075
DD3	25	10	3.0	333.3	30	5.0	0.150
DD4	25	10	3.0	333.3	30	7.5	0.225
DD5	25	10	3.0	333.3	30	10.0	0.300
LR1	25	10	3.0	333.3	30	5.0	0.150
LR2	25	10	3.0	333.3	30	5.0	0.150
LR3	25	10	3.0	333.3	30	5.0	0.150
LR4	25	10	3.0	333.3	30	5.0	0.150
Null				333.3	30		

^1^ the surface of area of pervious concrete is 0.09 m^2^, PZ refers to the TiO_2_ particle size, D refers to the TiO_2_ dosage, SD refers to the TiO_2_ spraying amount, DD refers to the dispersant dosage, LR refers to the light source. Null refers to control group.

**Table 3:** Ultraviolet irradiation intensity of ultraviolet lamp and sunlight.

Light Source	UV Height 135 mm	UV Height 180 mm	UV Height 225 mm	UV Height 270 mm	Sunlight
Ultraviolet irradiation intensity (μW/cm^2^)	105.1	48.4	31.8	19.1	104.9
